# Accelerated development of arthritis in mice lacking endothelial selectins

**DOI:** 10.1186/ar1770

**Published:** 2005-06-15

**Authors:** Jeffrey H Ruth, M Asif Amin, James M Woods, Xiaodong He, Sharon Samuel, Nengjun Yi, Christian S Haas, Alisa E Koch, Daniel C Bullard

**Affiliations:** 1 Northwestern University Feinberg School of Medicine, Chicago, Illinois, USA; 2 University of Michigan Medical School, Ann Arbor, Michigan, USA; 3 Midwestern University, Downers Grove, Illinois, USA; 4 Department of Genetics, University of Alabama at Birmingham, Birmingham, Alabama, USA; 5 Department of Biostatistics, University of Alabama at Birmingham, Birmingham, Alabama, USA; 6 Veteran's Administration Chicago Health Care Medical Center, Lakeside Division, Chicago, Illinois, USA; 7 Ann Arbor Veteran's Administration, Ann Arbor, Michigan, USA

## Abstract

The selectins, along with very late antigen-4 and CD44, have been implicated in mediating leukocyte rolling interactions that lead to joint recruitment and inflammation during the pathogenesis of rheumatoid arthritis. Previously, we showed that P-selectin deficiency in mice resulted in accelerated onset of joint inflammation in the murine collagen-immunized arthritis model. Here, we report that mice deficient either in E-selectin or in E-selectin and P-selectin (E/P-selectin mutant) also exhibit accelerated development of arthritis compared with wild type mice in the CIA model, suggesting that these adhesion molecules perform overlapping functions in regulating joint disease. Analyses of cytokine and chemokine expression in joint tissue from E/P-selectin mutant mice before the onset of joint swelling revealed significantly higher joint levels of macrophage inflammatory protein-1α and IL-1β compared to wild-type mice. IL-1β remained significantly increased in E/P-selectin mutant joint tissue during the early and chronic phases of arthritis. Overall, these data illustrate the novel finding that E-selectin and P-selectin expression can significantly influence cytokine and chemokine production in joint tissue, and suggest that these adhesion molecules play important regulatory roles in the development of arthritis in E/P-selectin mutant mice.

## Introduction

Leukocyte recruitment from the vasculature into tissue in response to an inflammatory stimulus is a regulated process that requires both adhesion proteins and chemoattractant/activating molecules [1]. Leukocyte emigration occurs primarily from the postcapillary venules and involves a cascade of events including leukocyte rolling, firm adhesion and activation, transendothelial migration, and migration into tissue. Rolling is mediated principally by the selectins (E-, L-, and P-selectin) and their ligands, although other adhesion molecules such as α_4 _integrins, vascular cell adhesion molecule-1, and CD44 can mediate rolling of certain leukocyte subtypes [2-4]. The selectins share a common structure characterized by an amino-terminal calcium-dependent lectin binding domain, an epidermal growth factor-like domain, a series of repeats with similarities to complement binding proteins, a transmembrane segment, and a short cytoplasmic tail [2]. L-selectin is expressed on the majority of leukocytes and is shed from the cell surface following activation, whereas P-selectin and E-selectin are expressed on endothelial cells following activation by various inflammatory mediators. Unlike the other selectins, P-selectin is also found on activated platelets.

Much of the early information about the roles of selectins in initiating leukocyte rolling and recruitment has come from *in vitro *or *in vivo *studies using function-blocking monoclonal antibodies or other inhibitors. During the past 10 years, targeted mutations in the genes that encode these proteins have been generated in mice. Collectively, these studies suggest that the selectins, particularly E-selectin and P-selectin, play important and overlapping roles in leukocyte rolling. However, the majority of these studies have focused on their contributions in neutrophil-dependent, short-term inflammatory models, and less is known about their roles in the development of chronic inflammatory diseases [5].

Rheumatoid arthritis (RA) is a systemic immune disorder characterized by polyarticular joint inflammation and destruction [6]. Increased expression of E-selectin and P-selectin has been observed in inflamed joints from RA patients, with several studies showing significant elevations in soluble selectins in the serum of patients with active disease [7-10]. In addition, several anti-inflammatory drugs have been shown to decrease the expression of E-selectin and P-selectin, as well as that of other adhesion molecules, in joint tissue from patients undergoing remission [7]. These findings suggest that the selectins may play an important role in the initiation and/or progression of joint inflammation during RA. However, investigations in animal models have provided inconsistent results concerning the role of selectins in the development of arthritis. For example, antibodies to E-selectin but not P-selectin inhibited adjuvant-induced arthritis in rats [11], whereas *Staphylococcus*-induced arthritis was diminished in P-selectin mutant mice and in mice treated with antibodies to L-selectin [12]. Previously, we reported that P-selectin mutant mice exhibited accelerated development of joint inflammation in the collagen-immunized arthritis (CIA) model compared with wild-type mice [13]. The severity of arthritis and the circulating levels of anti-type II collagen antibodies were also increased in P-selectin mutant mice. Further investigations suggested that this effect may result from alterations in leukocyte trafficking and/or cytokine production in lymphoid organs or joint tissue in these mice during the initiation of CIA [13].

In the present study, we analyzed both E-selectin mutant and E/P-selectin mutant mice in the CIA model. We observed that each of these mutants showed faster onset and more severe arthritis compared to wild-type mice, similar to our earlier studies of P-selectin deficient mice in the CIA model [13]. E/P-selectin mutants, along with E-selectin mutant mice, exhibited the most severe arthritis phenotype. Analyses of both cytokine and chemokine levels in joints from E/P-selectin mutant mice undergoing arthritis revealed elevated production of macrophage inflammatory protein (MIP)-1α and IL-1β compared with wild-type mice. Elevated joint IL-1β levels were consistently observed in the E/P-selectin mutants compared with wild-type mice throughout the course of the disease compared with nonimmunized mice.

## Materials and methods

### Mice

Mice with null mutations for E-selectin and E/P-selectin were generated by gene targeting in 129/Sv embryonic stem cells, as described previously [14]. The selectin mutations were then backcrossed into the CIA susceptible DBA-1/J strain (Jackson Laboratory, Bar Harbor, ME, USA) for five generations (N5). Inbred DBA-1/J mice were used as controls for all experiments. E/P-selectin mutant mice were kept on antibiotics (neomycin sulfate, tetracycline, and trimethoprim) until 2 weeks before the induction of CIA in order to inhibit the development of mucocutaneous infections, which commonly occur in these mice [15]. All mice were kept in pathogen-free conditions with regular health surveillance.

### Ethical use of animals

The University of Michigan's Unit for Laboratory Animal Medicine is an AAALAC (Association for Assessment and Accreditation of Laboratory Animal Care) accredited facility. Treatment of animals in this study was approved by ethics committees located initially at Northwestern University and later at the University of Michigan. Animal care at the Unit for Laboratory Animal Medicine is supervised by a veterinarian and operates in accordance with federal regulations. Mice were housed in sterile rodent micro-isolator caging with filtered cage tops in a specific pathogen-free environment to prevent infection. All efforts were made to reduce stress or discomfort in the animals used in these studies.

### CIA induction and scoring for CIA development

Chick type II collagen (Chondrex; LLC, Redman, WA, USA) was dissolved in 0.01 N acetic acid overnight at 4°C, and then emulsified with an equal volume of complete Freund's adjuvant (Difco, Detroit, MI, USA) to give a final concentration of 1 mg/ml. Male and female mice aged 8–12 weeks were injected intradermally at the base of the tail with 100 μl emulsified collagen on day 0 [13]. Arthritis was evaluated on a daily basis for 45 days, and then every other day until day 90 by an observer who was blinded to genotype. For these studies we used a scoring system identical to that used in our previously published CIA studies involving P-selectin and intercellular adhesion molecule-1 mutant mice [13,15], based on the methodology of Wooley and others [16-18]. Briefly, each joint was inspected and assessed for the severity of swelling using scores of 0 (normal appearance), 1 (mild), 2 (moderate), and 3 (severe), yielding a maximum score of 12 for each mouse. Swollen digits were noted but paws were only considered arthritic when the entire paw was inflamed for 2 consecutive days. The day of onset of arthritis was recorded for each mouse. During the chronic phase of the arthritis, paws and digits were inspected for distortion and manipulated to identify loss of flexion (ankylosis). Joints received an additional score of 3 for the presence of distortion and 3 for the presence of ankylosis (maximum additional score of 6 per joint). Daily severity scores were obtained from the addition of inflammation, distortion, and ankylosis scores. Scores for each mouse were used to derive a mean arthritis severity score for the group.

### Homogenization of joint tissue

E/P-selectin and wild-type mice were killed and both joints and serum collected at three periods during the course of disease: pre-arthritis (pre-CIA; days 16, 22, and 28), when no overt signs of arthritis were present; immediate post-arthritis onset (early-CIA), when mice were killed within 2 days of receiving a score of 3 for any joint; and chronic-CIA (day 80). Only hind joints were used in the study. Joints were removed directly below the hairline and snap frozen in liquid nitrogen. Joints were stored at -80°C before processing. Each joint was thawed on ice, weighed, and quickly homogenized on ice in 1–2 ml phosphate-buffered saline (PBS) containing a tablet of proteinase inhibitors (10 ml PBS/tablet; Boehringer Mannheim, Indianapolis, IN, USA). Homogenized tissues were centrifuged at 2000 *g *at 4°C for 10 min. Supernatants were stored at -80°C until analysis with ELISA.

### ELISA measurement of cytokines and chemokines in joint homogenate

ELISA reagents for cytokine measurement were obtained from R&D Systems (Minneapolis, MN, USA). Mouse cytokines were measured in joint homogenates, lymph node (LN) supernatants, and serum by ELISA using 96-well polystyrene plates, in accordance with the manufacturer's instructions. All homogenates were normalized to total protein and are presented as picogram or nanogram of cytokine per milligram of protein.

### ELISA measurement of anti-collagen antibodies

Mouse serum from E/P-selectin mutant, E-selectin mutant, and wild-type mice at 16 and 28 days after collagen immunization was analyzed for anti-collagen antibodies using a mouse IgG anti-type II collagen antibody assay kit, in accordance with the manufacturer's instructions (Chondrex, Inc., Redmond, WA, USA). ELISA samples were developed using a TMB substrate system and plates were read at 450 nm using an ELISA plate reader.

### Immunohistology

Frozen tissue (NL mouse and mouse CIA joints) were cut (approximately 7 μm) and immunoperoxidase stained using an avidin–biotin technique (Vector Laboratories, Burlingame, CA, USA) with all subsequent incubations being performed at 37°C in a humidified chamber. Slides were fixed in cold acetone for 20 min and then treated with 3% peroxidase in 0.1 mol/l Tris for 5 min to block endogenous peroxidase activity. Tissues were further blocked with 3% horse serum (in PBS) for 1 hour, and then incubated with goat anti-mouse IL-1β IgG (goat anti-mouse IL-1β; R&D Systems, Minneapolis, MN, USA) or goat anti-mouse MIP-1α IgG (goat anti-mouse/rat MIP-1α; Research Diagnostics Inc., Flanders, NJ, USA) or purified nonspecific goat IgG (Coulter, Miami, FL, USA). Tissue was washed twice in PBS, and a 1:200 dilution (in PBS) of anti-rat biotinylated antibody (Vector Laboratories) was added to the tissue sections and incubated for an additional 20 min. After a final washing (twice in PBS), slides were developed with a diaminobenzidine tetrahydrochloride substrate (Vector Laboratories) for 2 min at room temperature, rinsed in tap water, counter-stained with Harris' hematoxylin, and dipped in saturated lithium carbonate solution for bluing. Slides were examined for cellular immunoreactivity. Positive cells were identified by morphology.

### Isolation and culture of regional lymph nodes, spleen cells, preparation of supernatants, and serum collection

Draining inguinal LNs and spleen cells were collected at the time of joint harvest at pre-CIA (days 16, 22, and 28 after immunization), early-CIA (within 2 days of receiving a score of 3 for any joint), and chronic-CIA (80 days), and teased into single cell suspensions. Serum was collected from all mice at all time points, including day 0, pre-CIA, early-CIA, and chronic-CIA time points. LN cells were washed, counted, plated, and then cultured in RPMI containing 10% fetal bovine serum at 3 × 10^6 ^cells/ml in the presence or absence of 5 μg/ml chick type II collagen for 36 hours. After incubation, supernatants were collected by centrifugation and stored at -80°C. LN supernatants were then thawed and measured for cytokine levels. Data were combined for all time points and presented as wild-type or E/P-selectin LN cultures with or without stimulus. LN supernatant cytokine concentrations were normalized to pg/3 × 10^6 ^cells. Serum was collected when the animal was killed and measured for cytokine production, as described above.

### Statistical analysis

Three nonparametric statistical tests – Savage, Wilcoxon, and Kruskal–Wallis – were used to compare the incidence and severity of arthritis. These were calculated using SAS procedure NPAR1WAY (SAS Institute Inc., Cary, NC, USA). Similar statistical significance values were obtained with all three tests. Statistically significant differences in cytokine levels between groups was analyzed using the Student's independent t-test, with a threshold of *P *< 0.05.

## Results

### Accelerated development and increased severity of CIA in E- and E/P-selectin mutant mice

**Figure 1:**
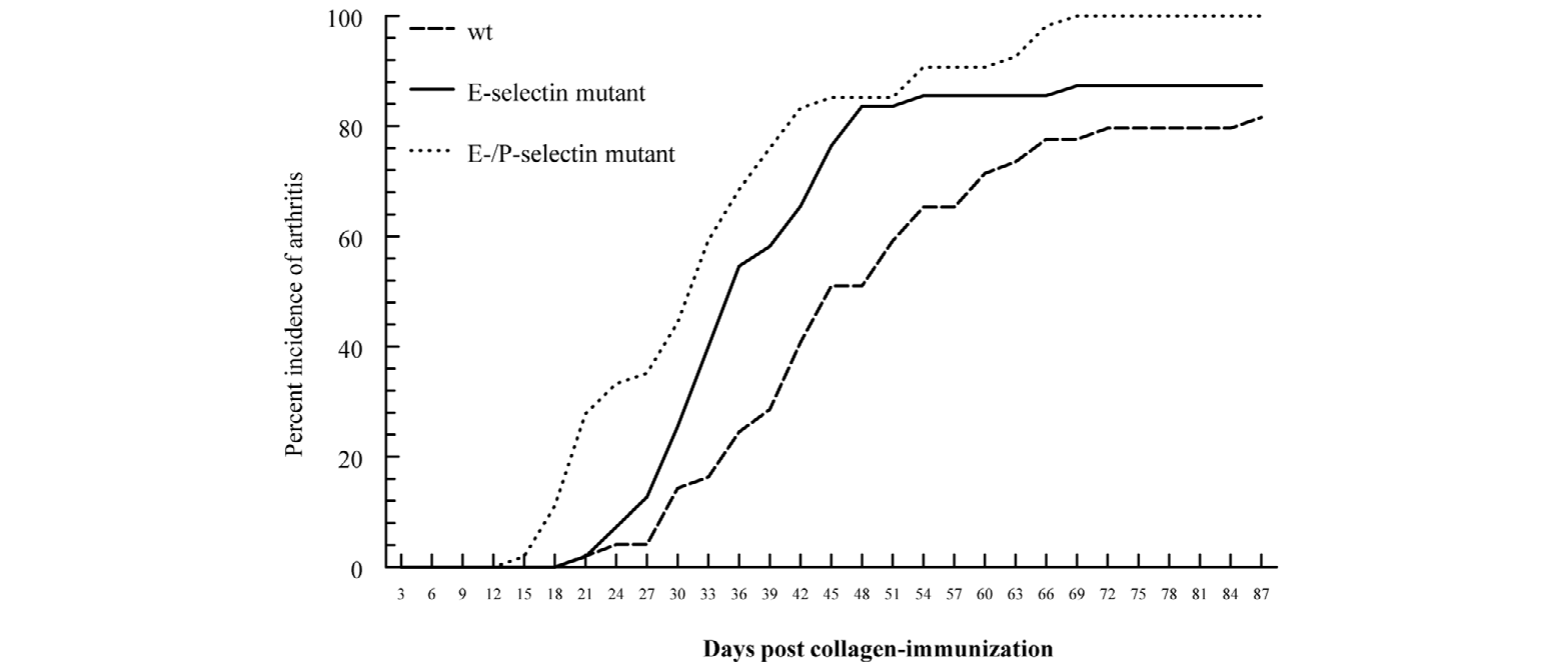
Incidence of CIA in E-selectin mutant, E/P-selectin mutant, and wild-type mice. E-selectin mutant (*n *= 56), E/P-selectin mutant (*n *= 54), and wild-type mice (*n *= 48) were immunized with chick type II collagen and assessed for the development of arthritis for 90 days by a blinded observer. The results are presented as the mean of three separate CIA experiments. The cumulative number of mice in each group is given as a percentage. CIA, collagen-immunized arthritis; wt, wild-type.

**Figure 2:**
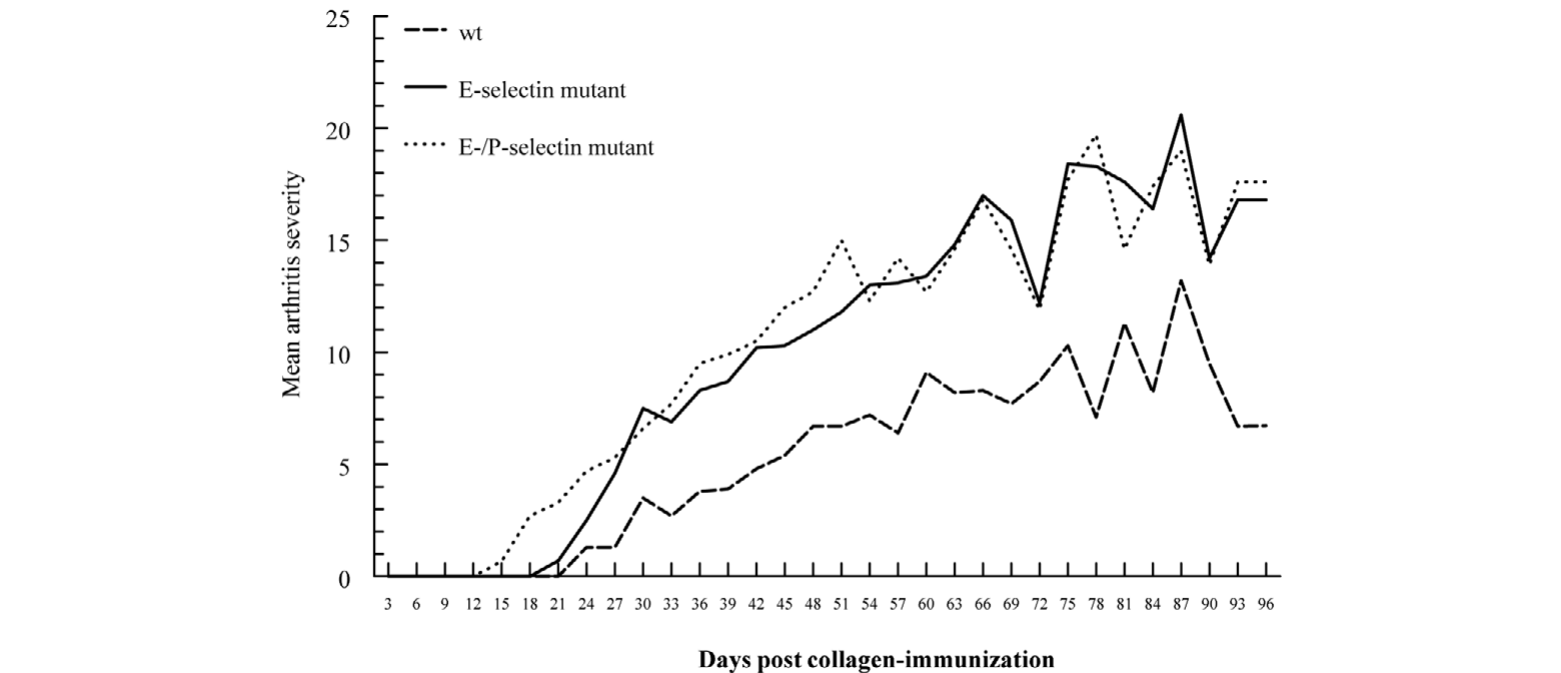
Arthritis severity in E-selectin mutant, E/P-selectin mutant, and wild-type mice. Arthritic paws were evaluated for the severity of inflammation and the presence of distortion and ankylosis as described in the Materials and method section. Daily severity scores were calculated from the combined inflammation, distortion, and ankylosis scores. Data are shown from three separate CIA experiments, and the mean arthritis severity is shown for each genotype. *P *< 0.05 for both E-selectin and E/P-selectin mutant versus wild type mice. wt, wild-type.

In three separate experiments, CIA was induced in E-selectin DBA/1J single mutant, E/P-selectin DBA/1J double mutant, and wild-type DBA/1J mice. Similar to our previously reported findings in P-selectin mutant mice [13], we observed a significant acceleration in the development of arthritis in both mutant groups (Fig. 1). The day of onset (mean ± standard error) was 45.2 ± 13.9 days for wild-type mice, 36.2 ± 11.0 days for E-selectin mutants, and 33.2 ± 11.4 days for E/P-selectin mutant mice (*P *< 0.01 for both E-selectin mutant and E/P-selectin mutant versus wild-type, as determined using the Kruskal–Wallis test). The E-/P-selectin mutants exhibited the most pronounced acceleratory effect, although the average day of onset was not significantly different compared with E-selectin mutants (*P *= 0.52). The incidence of arthritis was also increased in E/P-selectin mutant mice, with 100% showing signs of joint inflammation compared with 88% and 85% for E-selectin mutants and wild-type mice, respectively (Fig. 1). Additionally, both the E-selectin and E/P-selectin mutants had significantly higher average daily severity scores than did wild-type mice (Fig. 2). However, the severity scores were not significantly different between E-selectin and E/P-selectin mutant mice (*P *= 0.23 for E-selectin versus E/P-selectin).

### Increased joint expression of IL-1β and MIP-1α in collagen-immunized E-/P-selectin mutant mice prior to the onset of arthritis

**Figure 3:**
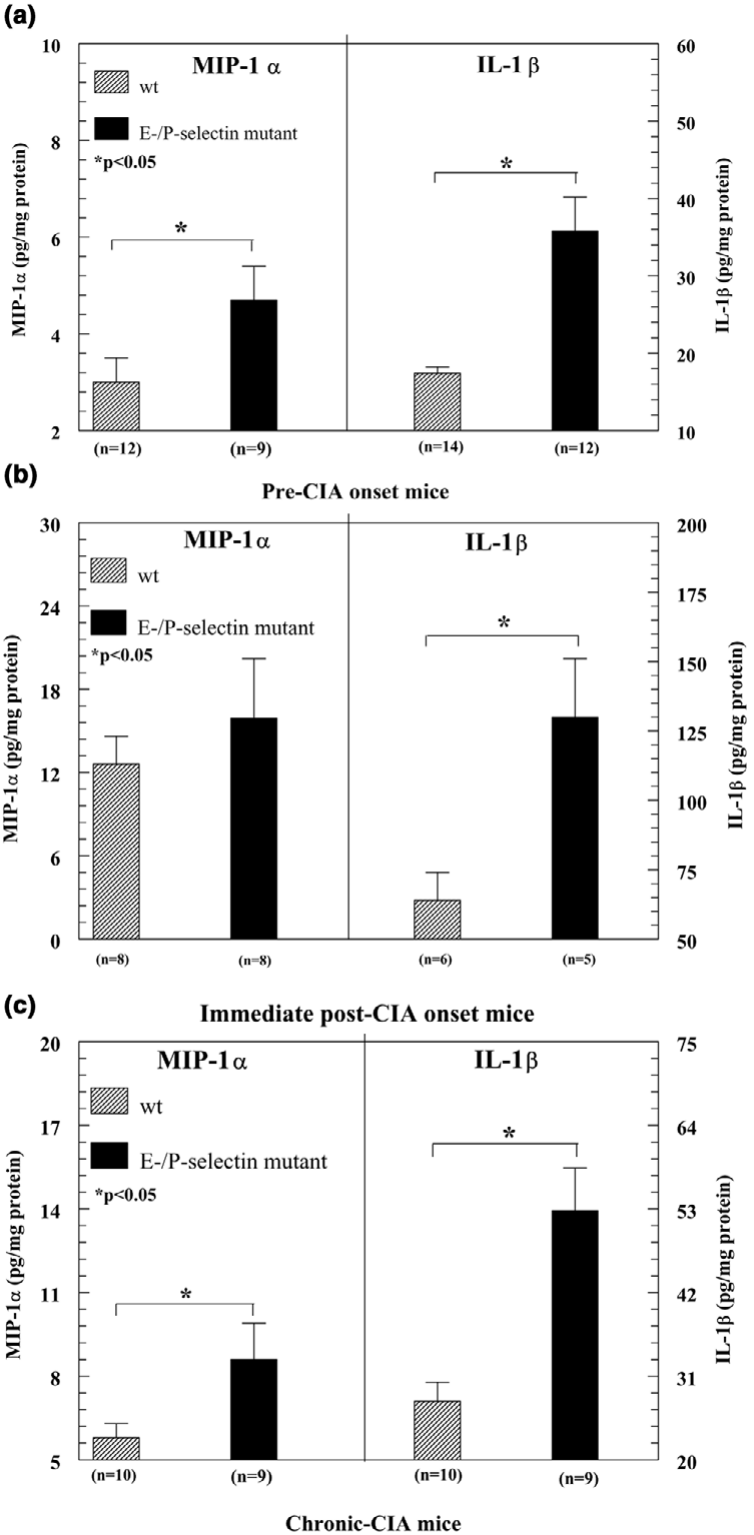
Cytokine expression in joint homogenates collected from pre-onset CIA mice. **(a) **Collagen-immunized E/P-selectin mutant mice exhibit increased MIP-1α and IL-1β in joint homogenates compared with immunized wild-type mice. **(b) **Joints from E-/P-selectin mutant mice during the early stages of arthritis show increased IL-1β expression compared with wild-type mice. **(c) **Chronic (day 80 collagen-immunized) E/P-selectin mutant mice show elevated levels of IL-1β and MIP-1α in their joint tissues compared with wild-type mice. Values are expressed as mean ± standard error of the mean. *P < 0.05. CIA, collagen-immunized arthritis; MIP, macrophage inflammatory protein; n, number of joints; wt, wild-type.

Altered cytokine production in synovial tissue appears to correlate with RA pathology [8]. Consequently, we investigated the hypothesis that mice that lack selectin molecules develop accelerated CIA as a result of altered cytokine expression. Because E/P-selectin mutant mice showed the strongest acceleration effect, we initially analyzed five different proinflammatory cytokine and chemokines levels in the joints of these mice before clinical signs of arthritis became obvious. Figure 3a shows the mean levels of MIP-1α and IL-1β expression in the joints of collagen-immunized pre-arthritic mice. Levels of both MIP-1α and IL-1β in the joint were elevated in E/P-selectin mutant mice. Table 1 shows the levels of the type 1 proinflammatory cytokines IL-12, tumor necrosis factor (TNF)-α, and MIP-2 in E/P-selectin mutants. In contrast to MIP-1α and IL-1β, we found no differences in these cytokines between immunized and/or nonimmunized wild-type and E/P-selectin mutant mice, indicating that IL-12, TNF-α, and MIP-2 do not directly correlate with the incidence or severity of CIA. We also measured joint levels of MIP-1α in nonimmunized wild-type and E/P-selectin mutant mice (data not shown) and observed no significant differences in basal levels of MIP-1α expression (values expressed as mean ± standard error; wild-type: 4.1 ± 0.5; E/P-selectin mutant: 4.1 ± 0.6). Serum was also collected from pre-arthritic mice. E-/P-selectin mutant mice had increased levels of IL-1β 22 days after immunization compared with wild-type mice (values expressed as mean ± standard error; wild-type: 54.5 ± 1.8 pg/ml; E/P-selectin mutant: 74.5 ± 6.4 pg/ml; *P *< 0.05, data not shown). There was a trend toward higher serum IL-1β in E/P-selectin mutants at days 16 and 28, but this was not statistically significant (data not shown).

### Elevated production of IL-1β in joint tissue of mice with early arthritis

We next analyzed cytokine levels in the joints of wild-type and E/P-selectin mutant mice showing the first signs of paw inflammation. Early-CIA E/P-selectin mutant mice exhibited significantly elevated levels of IL-1β but not of MIP-1α (Fig. 3b) or MIP-2 (Table 1) in their joint homogenates compared with wild-type mice. These findings indicate that in early-onset arthritic joints, expression of E-selectin and P-selectin can significantly influence the production of IL-1β but not of MIP-1α and MIP-2. As shown in Table 1, we found no differences between wild-type and E/P-selectin mutant mice in concentrations of IL-12 or MIP-2 within the joint, although we did observe increases in MIP-2 in the early-CIA joint tissue compared with the pre-arthritic samples. Hence, MIP-2 appears to be important in the development of CIA but it does not correlate with the acceleration in inflammation seen in E/P-selectin mutant mice. TNF-α levels in these joints were below the limit of detection.

### Elevated production of IL-1β and MIP-1α in chronically inflamed joint tissue

In order to examine the potential influence of selectins on the pattern of cytokine expression during the chronic phase of CIA, cytokines were measured in the joints of mice with CIA at day 80. Joint tissue (day 80) collected from E/P-selectin mutant mice had elevated levels of IL-1β and MIP-1α but not of MIP-2 compared with wild-type mice (Fig. 3c, Table 1). As shown in Table 1, IL-12 and MIP-2 levels in nonimmunized joints from both wild-type and E/P-selectin mutant mice were similar to those in immunized mice, suggesting MIP-2 or IL-12 are not pivotal cytokines in chronic-CIA. Consistent with the pre-CIA and early-CIA analyses, no significant concentrations of TNF-α were detected in the joints of wild-type or E/P-selectin mutant mice with chronic arthritis.

### Elevated levels of lymph node IL-1β and IL-2 from collagen-immunized E/P-selectin mutant mice

**Figure 4:**
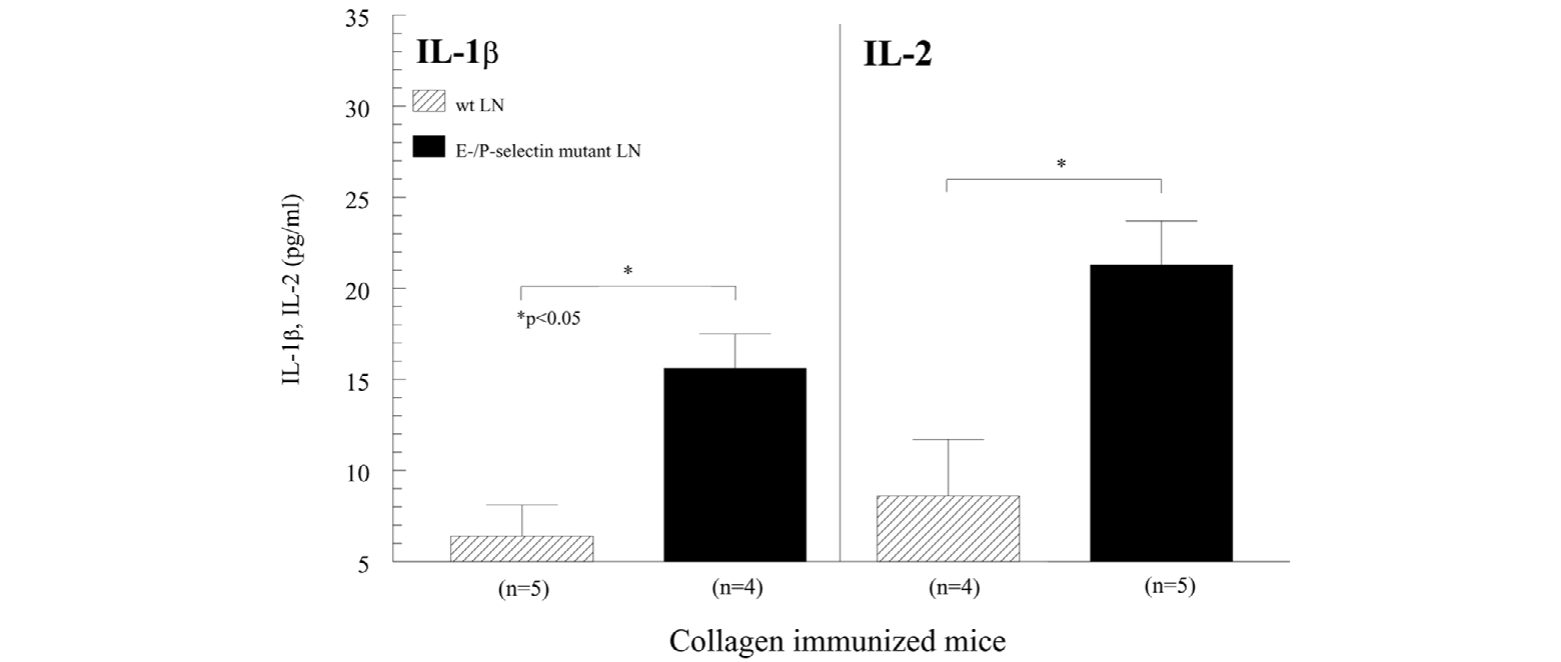
IL-1β and IL-2 expression from LN cells. IL-1β and IL-2 expression was increased in LN cell supernatants isolated from collagen-immunized E/P-selectin mutant mice compared with immunized wild-type mice. Pre-onset, post-onset, and chronic arthritis time points were combined. LN supernatants were stimulated with 5 μg/ml chick II collagen. Values are expressed as mean ± standard error of the mean. *P < 0.05. LN, lymph node; n, number of separate cultures; wt, wild-type.

In order to determine whether the systemic immune response is affected by deletion of these selectin molecules, we measured cytokine expression from collagen-stimulated and nonstimulated draining inguinal LN cells obtained from immunized and nonimmunized wild-type and E/P-selectin mutant mice. E/P-selectin mutant mice had elevated IL-1β and IL-2 production from inguinal LN cells compared with collagen-immunized wild-type mice (Fig. 4), indicating that deleting selectin genes can affect the systemic immune response during CIA development. This is consistent with the joint cytokine measurements, showing increased IL-1β production in pre-CIA onset, post-CIA onset, and chronic-CIA E-/P-selectin mutant mice (Fig. 3).

### Increased IL-1β production in serum and joints of nonimmunized E/P-selectin mutant mice

**Figure 5:**
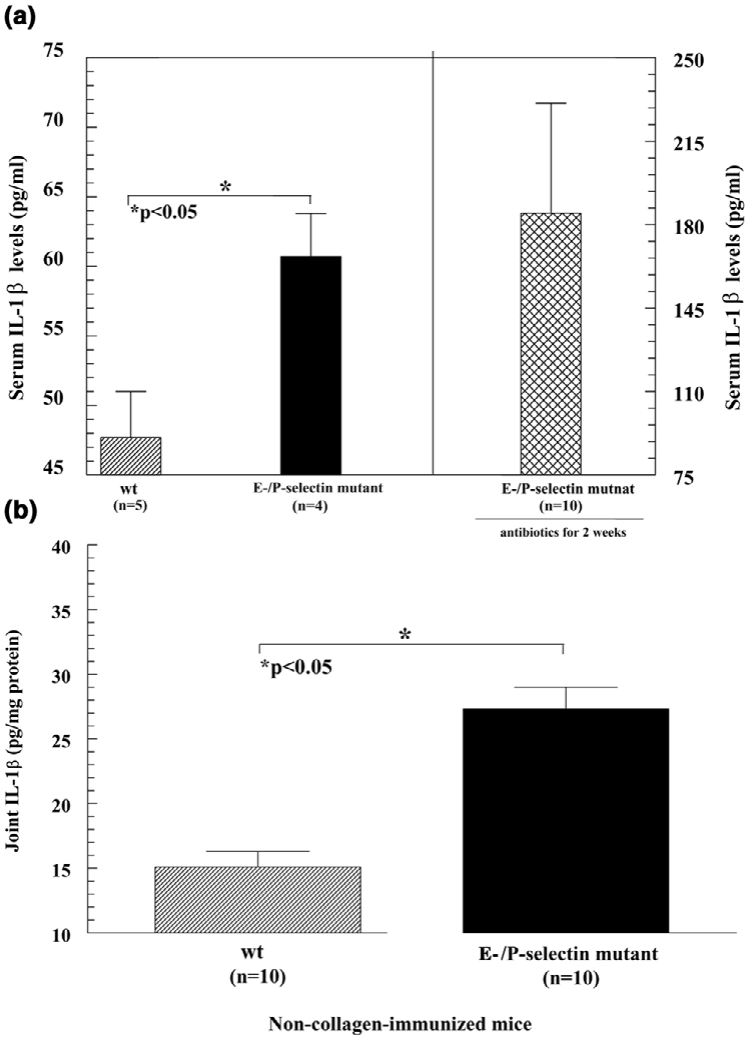
IL-1β expression in serum and joint tissue from non-collagen-immunized mice. **(a) **IL-1β was significantly elevated in serum from nonimmunized E/P-selectin mutant compared with nonimmunized wild-type mice. Serum from E/P-selectin mutant mice kept on antibiotics for 2 weeks also had elevated serum IL-1β levels, indicating that the basal overexpression of IL-1β in nonimmunized E/P-selectin mutant mice is not due to bacterial infections. **(b) **IL-1β was also significantly increased in joint homogenates from nonimmunized E/P-selectin mutant compared with nonimmunized wild-type mice. Values are expressed as mean ± standard error of the mean. *P < 0.05. n, number of mice (panel (a) or number of joints (or mice for serum; panel (b); wt, wild-type.

To investigate the possibility that mice lacking selectin molecules are predisposed to develop an accelerated inflammatory response, we analyzed basal proinflammatory cytokine concentrations in the serum and joints of nonimmunized wild-type and E/P-selectin mutant mice. We found increased serum levels of IL-1β but not of MIP-1α (data not shown for MIP-1α) in E/P-selectin mutant compared with wild-type mice (Fig. 5a). In addition, there was approximately a twofold increase in the concentration of IL-1β in joint tissue from nonimmunized E/P-selectin mutant mice compared with wild-type mice (Fig. 5b).

We examined nonimmunized mouse serum for IL-1β to test whether E/P-selectin mutant mice may be primed to elicit an inflammatory response to administered antigen by overexpressing basal levels of IL-1β. As shown in Fig. 5a, nonimmunized E/P-selectin mutant mice had significantly elevated levels of IL-1β in their serum compared with wild-type mice. In order to rule out the possibility that the elevated serum IL-1β levels in E/P-selectin mice were due to infections, we kept nonimmunized E-/P-selectin mutant mice on antibiotics for 2 weeks and then examined serum IL-1β levels. As shown in Fig. 5a, serum IL-1β levels were not lowered by antibiotic treatment but actually increased. This shows that the elevated IL-1β in the serum of E/P-selectin mutant mice is not due to infection.

### Serum and joint IL-1 receptor antagonist protein levels are not elevated in E/P-selectin mutant compared with wild-type mice

**Figure 6:**
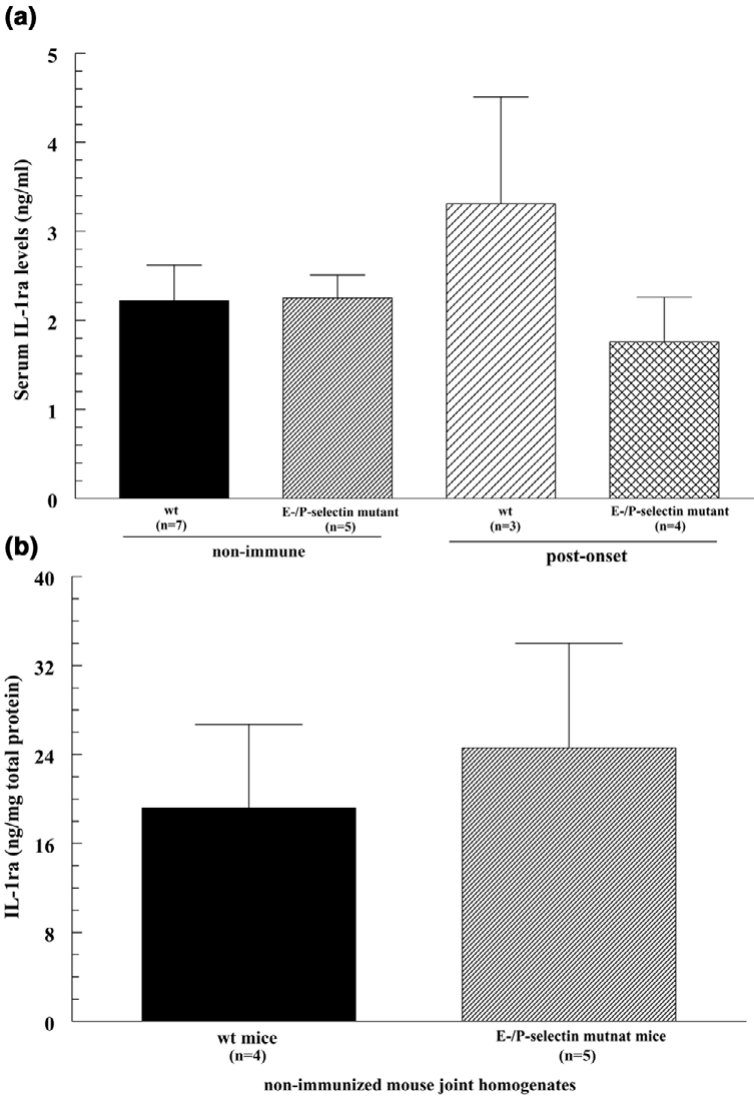
IL-1ra expression in serum and joint tissue in wild-type and E/P-selectin mutant mice. **(a) **IL-1ra was not significantly elevated in serum from nonimmunized and post-onset CIA E/P-selectin mutant mice compared with serum from wild-type mice. **(b) **IL-1ra levels were higher in the joints compared with serum of nonimmunized wild-type and E/P-selectin mutant and wild-type mice, but no difference in IL-1ra levels was found in joint homogenates from nonimmunized E/P-selectin mutant and nonimmunized wild-type mice. Values are expressed as mean ± standard error of the mean. *P < 0.05. IL-1ra, IL-1 receptor antagonist; n, number of mice (panel (a) or number of joints (or mice for serum; panel (b); wt, wild-type.

We examined the serum of nonimmunized and post-CIA onset E/P-selectin mutant and wild-type mice for IL-1 receptor antagonist (IL-1ra) expression by ELISA. We found that both wild-type and selectin mutant mice had approximately 2 ng/ml IL-1ra in their serum, but we did not find any statistically significant differences between wild-type and selectin mutant mice. Similar results were obtained from the serum of post-CIA onset mice (Fig. 6a). We also examined the joints of wild-type and E/P-selectin mutant mice for IL-1ra expression normalized to total protein levels. Figure 6b shows that IL-1ra was detected in higher amounts in whole joint homogenates than in serum of both nonimmunized wild-type and E/P-selectin mutant mice, but we did not find differences in IL-1ra expression between nonimmunized wild-type and E/P-selectin joint homogenates, suggesting selectin deficiency does not significantly alter IL-1ra expression in the serum or joints.

### Mouse serum IgG anti-collagen antibody concentration after collagen immunization is not increased in selectin mutant compared with wild-type mice in pre-CIA onset mice

**Figure 7:**
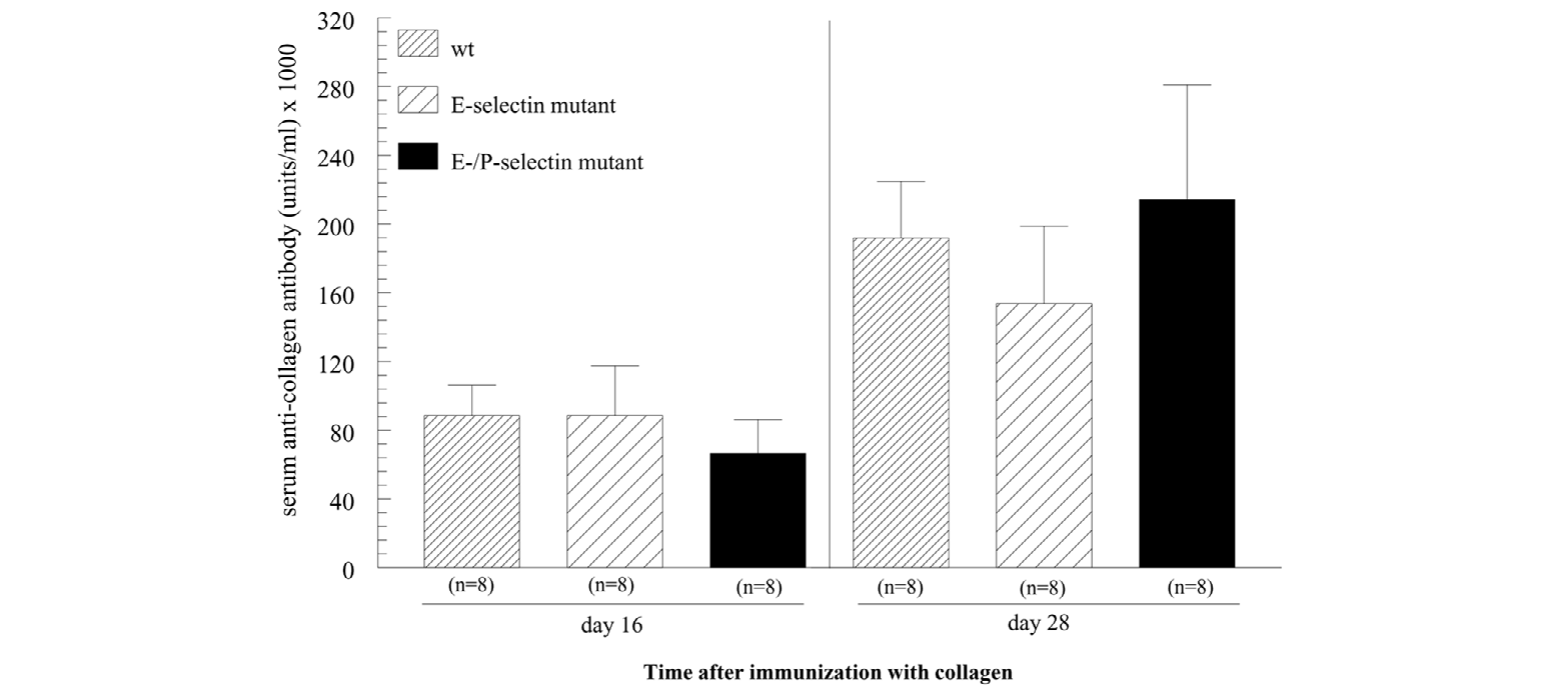
Endothelial selectin deficiency does not influence anti-collagen antibody production in collagen immunized mice. Mouse serum anti-collagen IgG antibody concentration is not altered in single E-selectin and E/P-selectin mutant mice compared with wild-type mice at days 16 and 28 after collagen immunization. Wild-type and selectin mutant mice were administered collagen, and then at days 16 and 28 mice were bled and serum was measured for anti-collagen IgG. Although anti-collagen IgG was elevated in all the groups at day 28 compared with day 16, there were no differences in anti-collagen IgG between groups at the time points examined. Values are expressed as mean ± standard error of the mean. wt, wild-type.

To determine whether selectin mutant mice may have altered responses to collagen immunization compared with wild-type mice, we examined murine serum anti-collagen antibody concentrations at days 16 and 28 post-CIA immunization in wild-type, E-selectin mutant, and E/P-selectin mutant mice not showing clinical signs of arthritis. As shown in Fig. 7, wild-type and selectin mutant mice had elevated serum levels of anti-collagen IgG at day 28 compared with day 16 mice, but we did not find significant differences in anti-collagen antibody concentrations between wild-type, E-selectin, and E/P-selectin mutant mice. This suggests that selectin deficiency does not alter responses to collagen during the initiation of arthritis.

### Immunohistochemistry for IL-1β and MIP-1α in synovial tissue of E/P-selectin mutant mice with CIA

**Figure 8:**
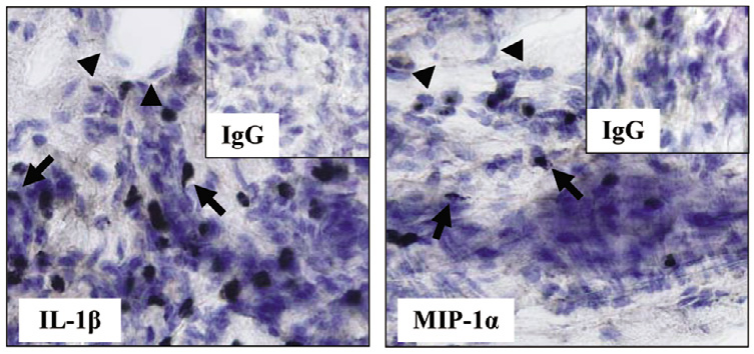
Macrophages stain positive for IL-1β and MIP-1α in the joints of nonimmunized E/P-selectin mutant mice. Immunohistochemistry for IL-1β and MIP-1α in synovial tissue of E/P-selectin mutant CIA mice. Staining for both cytokines showed clear immunopositivity on macrophages (arrows), whereas ST endothelial cells (arrowheads) remained negative. Original magnification: 400×. CIA, collagen-immunized arthritis; MIP, macrophage inflammatory protein; wt, wild-type.

To determine the cell types responsible for elevated IL-1β and MIP-1α in the joints of E/P-selectin deficient mice, we performed immunohistology for both cytokines using goat anti-mouse IL-1β or goat anti-mouse/rat MIP-1α. The staining clearly shows immunopositivity on macrophages (arrows), whereas ST endothelial cells (arrowheads) remained negative (Fig. 8).

## Discussion

Studies in both RA patients and in animal models of arthritis strongly implicate adhesion molecules as critical mediators in both the initiation and maintenance of joint inflammation [7]. Multiple adhesion molecule pathways appear to mediate leukocyte interactions, particularly rolling, within the synovial vasculature. Support for this model comes from studies showing loss or inhibition of CD44 and VLA-4 (very late antigen-4) interactions with their ligands that can reduce leukocyte rolling interactions and inhibit joint inflammation in animal models of arthritis [19,20]. However, conflicting results have been published with regard to the roles of E-selectin and P-selectin in the development of joint inflammation in different animal models [11-13,21]. Our previous and current studies in the CIA model show that leukocyte rolling via E-selectin and P-selectin is not required for the initiation of arthritis, and that loss of one or both of these adhesion molecules significantly accelerates the development and severity of joint inflammation. In the present study there was no significant difference in day of onset, number of mice showing arthritis, or severity between the single E-selectin and the E/P-selectin mutant mice but, overall, selectin mutant mice experienced accelerated CIA development compared with wild-type mice. This may be explained by a limit on how quickly a mouse can actually develop CIA, even with loss of both E-selectin and P-selectin. These factors may include the time for immunization to occur, loss of tolerance to collagen, and immune complex deposition in the joints. Also, once destructive processes such as bone erosion start in the joint, individual scores do not change significantly.

Accelerated development and/or increased severity of CIA in mice have previously been reported where cytokine expression or activity has been altered [17,18,22,23]. Therefore, we investigated whether the loss of E-selectin or P-selectin affected the expression levels of five different cytokines and chemokines that are implicated in the development of CIA. Our analyses in E/P-selectin mutant mice suggested that these adhesion molecules may influence the development of CIA by altering the production of MIP-1α and IL-1β. E/P-selectin mutant mice also produced significantly more IL-1β in their joints and serum than did wild-type mice at all of the time points examined, strongly implicating IL-1β in accelerated development and increased severity of CIA. These studies suggest that increased levels of IL-1β contribute to the accelerated development of arthritis in double selectin deficient mice.

Of significance, E/P-selectin mutant mice have elevated basal levels of IL-1β in their hind joints compared with wild-type mice, without collagen immunization. Therefore, mice lacking both E-selectin and P-selectin are seemingly primed to mount a robust inflammatory response in their joints. We also observed increased MIP-1α levels in pre-CIA onset and chronic-CIA mice, showing that selectins may also regulate chemokine production. In order to rule out the possibility that E/P-selectin mutant mice have enhanced basal levels of IL-1β due to low-grade infections, we measured basal IL-1β levels in nonimmunized E/P-selectin mutant mice kept on antibiotics for 2 weeks. We found that IL-1β levels were not decreased in joints of E/P-selectin mutant mice, but actually increased. Therefore, it is likely that IL-1β is regulated differently in mice that lack selectin molecules, and that these differences are not due to infection.

We also measured basal levels of IL-1ra – the natural inhibitor of IL-1β – in the joints and serum of both wild-type and E/P-selectin mutant mice. We found substantial levels of IL-1ra in both nonimmunized wild-type and nonimmunized E/P-selectin mutant mouse joints and serum, but we did not find elevated levels of IL-1ra in E/P-selectin mutant compared with wild-type mice. We also measured serum of post-onset CIA wild-type and E/P-selectin mutant mice for IL-1ra, and again observed no significant differences between wild-type and selectin deficient groups. These findings are in agreement with previous reports that have shown TNF-α, but not IL-1β, to be a significant *in vivo *and *in vitro *stimulus for murine IL-1ra production [24]. Although TNF-α is a well established cytokine in RA pathology, TNF-α was not detected in any mice at any of the time points measured in the study. This cytokine may not be necessary for the development of arthritis in this model, because a study recently reported that severe CIA occurs in TNF-α deficient mice [25]. It is currently unknown whether similar mechanisms are at work in single selectin deficient mice, and whether other inflammatory mediators may be responsible for the accelerated CIA seen in these mice. We also measured murine anti-collagen antibodies in the serum of day 16 and day 28 pre-onset CIA wild-type, E-selectin, and E/P-selectin mutant mice to determine whether anti-collagen antibodies may be regulated differently in selectin deficient and wild-type mice. We showed that anti-collagen antibody levels were generally increased at day 28 compared with day 16 mice, but we observed no significant differences between wild-type and E/P-selectin mutant mice, suggesting that anti-collagen antibody is not a determining factor in the accelerated CIA development seen in the E/P-selectin mutant mice.

Interestingly, we did not observe significant differences in the cellular infiltrate by immunohistology in the joints of the wild-type, E-selectin, and E/P-selectin mutant mice. In our hands, the cell composition was similar between groups, but only the severity and timing of the inflammatory response was altered in mice lacking selectins, probably due to exaggerated and early IL-1β production. This is concomitant with the elevated blood leukocyte counts observed in P-selectin and E/P-selectin mutant mice compared with wild-type mice, suggesting that the elevated leukocytes in selectin deficient mice may be the cause of accelerated CIA. However, E-selectin mutant mice do not have elevated blood leukocyte counts, but they also exhibit the accelerated CIA phenotype [14]. Therefore, the increased cytokine production in the E/P-selectin mutant mice cannot be fully explained by elevated white blood cell numbers.

Further work is necessary to determine whether similar alterations occur in other adhesion molecule deficient mice and to investigate the possibility that these molecules regulate the development of arthritis via a common pathway. An alternative mechanism may involve the disruption of regulatory leukocyte recruitment, including T regulatory cells, into the joints of selectin deficient mice during the initial phases of arthritis. A number of studies have shown that E-selectin and P-selectin selectively mediate rolling of specific leukocyte subsets, such as T-helper-1 cells, but not T-helper-2 cells [26], although their contributions to T regulatory cell recruitment remain to be defined. We are currently examining the roles of the selectins in regulatory and effector T cell recruitment during the development of arthritis in this model. It is also possible that the lack of certain selectin molecules may result in an overexpression of other adhesion molecules and/or selectins that may selectively recruit IL-1β producing monocytes to the joints. This would result in the observed elevated monokine production in mice lacking E-selectin and P-selectin.

Several studies have identified IL-1β as a potent proinflammatory cytokine in animal models of RA, and it is likely that this cytokine contributes to the more severe disease phenotype observed in the double mutants. For example, van den Berg and coworkers [27] showed that a combination of antibodies to IL-1α and IL-1β given before the onset of arthritis completely prevented murine CIA. In addition, exogenously administered IL-1β was found to potentiate both murine and rat CIA [28,29]. In another study, IL-1β was shown to be critical for accelerated development of CIA, and expression of this cytokine correlated with increased incidence and earlier disease onset [30]. It was previously reported that mice overexpressing IL-1ra exhibited reduced incidence and severity of CIA, whereas mice deficient in the IL-1ra gene showed accelerated development of arthritis with increased severity [31]. In other studies, BALB/c mice lacking IL-1ra spontaneously developed chronic inflammatory polyarthropathy, with joint pathology closely resembling that of RA [32]. These findings clearly underscore the importance of IL-1β in arthritis development.

Our findings that MIP-1α levels were elevated in E/P-selectin mutant mice are concordant with previous reports that MIP-1α is proinflammatory in RA and CIA [33-35]. Increased MIP-1α in the joints of pre-arthritic E/P-selectin mutant mice may also contribute to increased IL-1β, because MIP-1α is chemotactic for monocytes, which produce significant amounts of IL-1β [36]. These findings are consistent with histology studies showing that macrophages are the significant producers of both MIP-1α and IL-1β in the joints of E/P-selectin mutant mice with CIA. This suggests that macrophages in E/P-selectin mutant mice may be altered in some way to overproduce both MIP-1α and IL-1β, or that that these monocytes are specifically recruited to the joints of selectin deficient mice during CIA development. Interestingly, we did not observe significant expression of TNF-α in joint tissue in our study. It is possible that TNF-α is present in CIA but is expressed only transiently before the onset of disease. Previous studies of murine CIA have indicated that TNF-α is expressed very early in the disease, before IL-1β [37], and is tightly regulated, whereas IL-1β expression is not [24].

## Conclusion

We have observed accelerated development and increased severity of CIA in P-selectin, E-selectin, and E/P-selectin mutant mice, suggesting that these adhesion molecules play important regulatory roles in the development of arthritis. In addition, we found that E/P-selectin mutant mice show significantly increased expression of IL-1β in the joints and serum under basal conditions, increased IL-1β and MIP-1α expression in joint tissue at several time points during the development of CIA, and increased LN cell production of both IL-1β and IL-2. This altered immune response may be due to built in compensatory mechanisms or disrupted homeostasis, resulting in either monocyte over-recruitment or overstimulation of tissue macrophages in E/P-selectin mutant mice. Alternatively, these findings may suggest a novel function for these selectins in regulating expression of cytokines and chemokines in joint and other tissues during chronic inflammation.

## Abbreviations

CIA = collagen-immunized arthritis; ELISA = enzyme-linked immunosorbent assay; IL = interleukin; IL-1ra = interleukin-1 receptor antagonist; LN = lymph node; MIP = macrophage inflammatory protein; PBS = phosphate-buffered saline; RA = rheumatoid arthritis; TNF = tumor necrosis factor.

## Competing interests

The author(s) declare that they have no competing interests.

## Authors' contributions

JHR, the first author, designed and developed all aspects of the study. MAA and JMW assisted in the animal scoring and cytokine measurements. XH assisted with the development and generation of the selectin deficient mice, and performed the anti-collagen ELISA. SS, of Dr Bullard's group, immunized mice and conducted the scoring. NY performed the necessary statistical analyses. CSH performed the immunohistology work. AEK, a senior author, was instrumental in the design and development of this project, and generously offered her expertise. DCB, a co-senior author, developed, designed, and generated the selectin deficient mice, as well as supplied murine joints and serum that made this study possible. DCB was also responsible for generating CIA development in selectin deficient mice, and offered valuable advice.

## Figures and Tables

**Table 1:** Cytokine levels

Cytokine	Genotype	Cytokine level	*P *(versus E/P-selectin mutant)	Number of observations
IL-12	Wild-type			
	Nonimmunized	2.2 ± 0.2 pg/mg	>0.05	12
	Pre-onset	ND	ND	0
	Post-onset	1.6 ± 0.2 pg/mg	>0.05	8
	Chronic	2.6 ± 0.6 pg/mg	>0.05	8
				
	E/P-selectin mutant			
	Nonimmunized	5.7 ± 3.1 pg/mg		10
	Pre-onset	ND		0
	Post-onset	1.7 ± 0.2 pg/mg		8
	Chronic	2.3 ± 0.5 pg/mg		8

TNF-α^a^	Wild-type			
	Nonimmunized	<1 pg/mg	NS	10
	Pre-onset	<1 pg/mg		13
	Post-onset	<1 pg/mg		8
	Chronic	<1 pg/mg		8
				
	E/P-selectin mutant			
	Nonimmunized	<1 pg/mg		10
	Pre-onset	<1 pg/mg		11
	Post-onset	<1 pg/mg		7
	Chronic	<1 pg/mg		8
				
MIP-2	Wild-type			
	Nonimmunized	2.2 ± 0.4 pg/mg	>0.05	12
	Pre-onset	1.9 ± 0.9 pg/mg	>0.05	12
	Post-onset	5.3 ± 0.9 pg/mg	>0.05	8
	Chronic	1.4 ± 0.1 pg/mg	>0.05	10
				
	E/P-selectin mutant			
	Nonimmunized	1.6 ± 0.2 pg/mg		10
	Pre-onset	2.4 ± 1.8 pg/mg		9
	Post-onset	5.2 ± 1.6 pg/mg		8
	Chronic	1.7 ± 0.5 pg/mg		9

Cytokines were not elevated in murine joint homogenates from nonimmunized E/P-selectin mutant mice, or from pre-CIA onset, post-CIA onset, and chronic-CIA (day 80) E/P-selectin mutant mice, compared with wild-type mice. Values for TNF-α are below the sensitivity of the assay. ELISA assay sensitivities were 2.5 pg/ml for IL-12, 5 pg/ml for TNF-α and IL-1β, and 1.5 pg/ml for MIP-2 and MIP-1α. Pre-onset group corresponds to combined values for day 16, day 22, and day 28 groups. All values are normalized to milligrams of total protein. CIA, collagen-immunized arthritis; MIP, macrophage inflammatory protein; ND, no data.
